# Supplementary material for: Systematic Characterization and Regulatory Role of lncRNAs in Asian Honey Bees Responding to Microsporidian Infestation
Source: Int J Mol Sci. 2023 Mar 20;24(6):5886. doi: 10.3390/ijms24065886 (PMC10058195; doi:10.3390/ijms24065886)
Supplement: Supplementary file 1 [file ijms-24-05886-s001.zip › Figure S1.pdf]

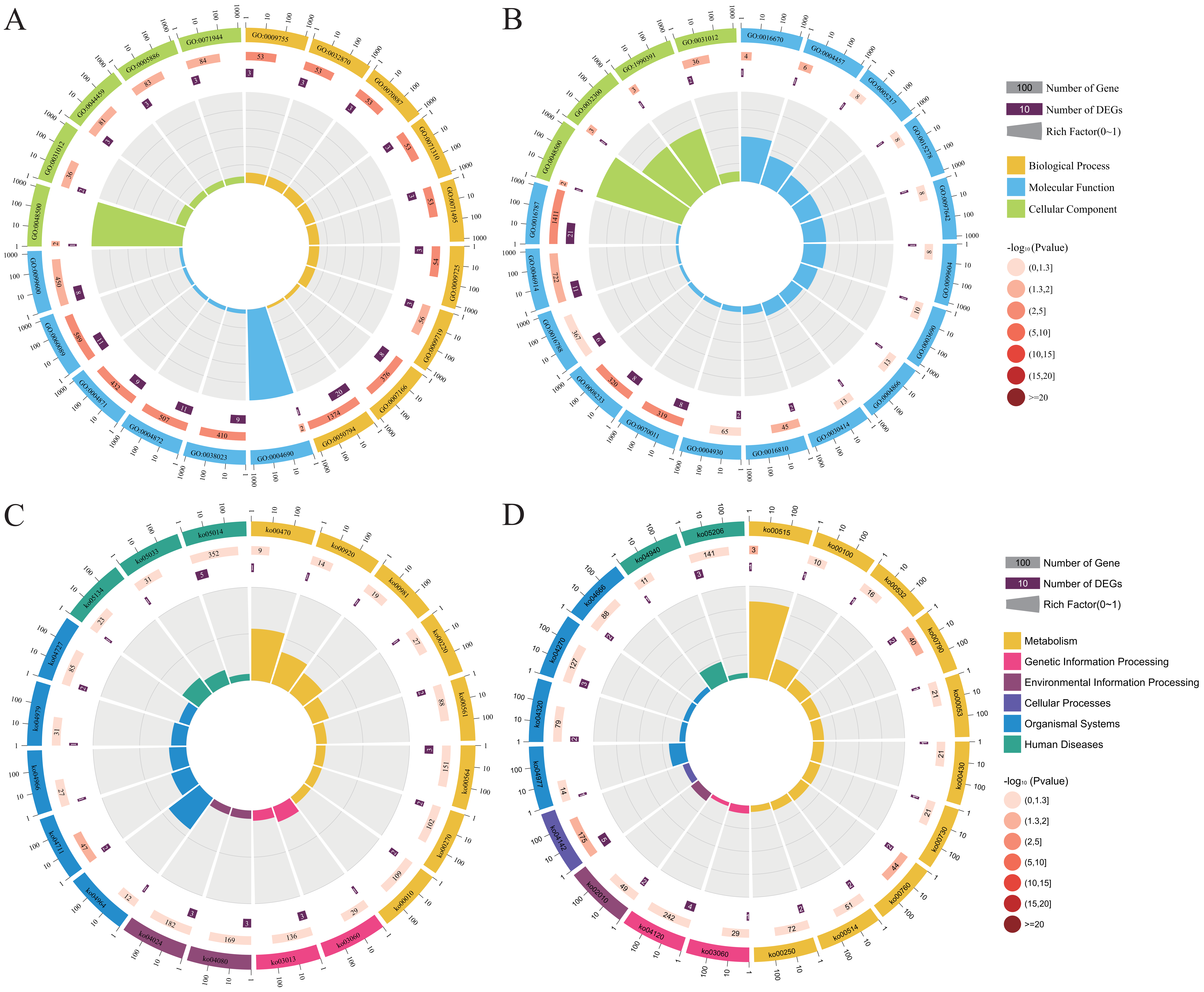

Figure S1. GO and KEGG analysis of DElncRNA co expressed genes in AcCK1 vs. AcT1 and AcCK2 vs. AcT2 comparison groups.
A: GO analysis of DElncRNA co expressed genes in AcCK1 vs. AcT1 comparison group;
B: GO analysis of DElncRNA co expressed genes in AcCK2 vs. AcT2 comparison group;
C: KEGG analysis of DElncRNA co expressed genes in AcCK1 vs. AcT1 comparison group;
D: KEGG analysis of DElncRNA co expressed genes in AcCK2 vs. AcT2 comparison group.
