# Supplementary material for: Systematic Characterization and Regulatory Role of lncRNAs in Asian Honey Bees Responding to Microsporidian Infestation
Source: Int J Mol Sci. 2023 Mar 20;24(6):5886. doi: 10.3390/ijms24065886 (PMC10058195; doi:10.3390/ijms24065886)
Supplement: Supplementary file 1 [file ijms-24-05886-s001.zip › Table S2.pdf]

Table S2. Summary of top 20 up-and down-regulated lncRNAs within AcCK1 vs. AcT1

LncRNAID	The FPKM value of AcCK1 vs. AcT1	The log_2 (Foldchange) of AcCK1 vs. AcT1	*P* value
XR_001767038.1	18.9000	12.4691	0.0000
TCONS_00047233	1.5000	10.5507	0.0005
XR_001766127.1	1.3867	10.4374	0.0000
XR_001765163.1	1.3133	10.3590	0.0198
TCONS_00029392	1.3033	10.3480	0.0302
XR_001765315.1	1.2133	10.2448	0.0000
TCONS_00006106	1.1233	10.1336	0.0293
TCONS_00029812	1.1200	10.1293	0.0298
XR_001765926.1	1.0100	9.9801	0.0231
TCONS_00004253	0.9400	9.8765	0.0079
XR_001767044.1	0.8200	9.6795	0.0210
TCONS_00012188	0.7733	9.5949	0.0276
XR_001765299.1	0.6800	9.4094	0.0018
TCONS_00032459	0.6233	9.2839	0.0204
TCONS_00017999	0.6200	9.2761	0.0207
XR_001765180.1	0.5600	9.1293	0.0374
TCONS_00022015	0.5567	9.1207	0.0048
TCONS_00019291	0.5167	9.0131	0.0041
TCONS_00019419	0.4600	8.8455	0.0356
XR_001765320.1	0.3967	8.6318	0.0096
TCONS_00042235	0.3167	-8.3068	0.0165
TCONS_00026623	0.3533	-8.4649	0.0166
TCONS_00019289	0.3733	-8.5443	0.0443
XR_001765993.1	0.4833	-8.9169	0.0094
XR_001764971.1	0.5133	-9.0038	0.0035
TCONS_00011721	0.5400	-9.0768	0.0041
TCONS_00046801	0.5400	-9.0768	0.0226
XR_001766966.1	0.5400	-9.0768	0.0349
TCONS_00037364	0.5600	-9.1293	0.0073
TCONS_00038004	0.5767	-9.1716	0.0445
XR_001765515.1	0.6167	-9.2683	0.0027
TCONS_00031155	0.7033	-9.4581	0.0409
TCONS_00001436	0.8900	-9.7977	0.0181
XR_001765500.1	0.9400	-9.8765	0.0490
XR_001766403.1	0.9933	-9.9561	0.0124

XR_001766879.1	1.1400	-10.1548	0.0276
XR_001765115.1	1.2433	-10.2800	0.0163
XR_001766607.1	1.3200	-10.3663	0.0132
XR_001765791.1	1.5400	-10.5887	0.0000
TCONS_00006288	1.7367	-10.7621	0.0244
