# Supplementary material for: Systematic Characterization and Regulatory Role of lncRNAs in Asian Honey Bees Responding to Microsporidian Infestation
Source: Int J Mol Sci. 2023 Mar 20;24(6):5886. doi: 10.3390/ijms24065886 (PMC10058195; doi:10.3390/ijms24065886)
Supplement: Supplementary file 1 [file ijms-24-05886-s001.zip › Table S3.pdf]

Table S3. Summary of top20 up-and down-regulated lncRNAs within AcCK2 vs. AcT2

LncRNAID	The FPKM value of AcCK2 vs. AcT2	The log_2 (Foldchange) of AcCK2 vs. AcT2	*P* value
TCONS_00029732	8.6100	13.0718	0.0457
TCONS_00018981	5.5933	12.4495	0.0352
XR_001767090.1	3.8933	11.9268	0.0128
TCONS_00024777	3.8800	11.9218	0.0255
TCONS_00022015	3.1133	11.6042	0.0020
XR_001765858.1	2.9033	11.5035	0.0013
TCONS_00042438	1.8900	10.8842	0.0351
TCONS_00045870	1.7667	10.7868	0.0089
TCONS_00007296	1.7467	10.7704	0.0085
XR_001765478.1	1.7100	10.7398	0.0059
TCONS_00007982	1.5567	10.6042	0.0494
XR_001766995.1	1.3700	10.4200	0.0010
TCONS_00023147	1.2633	10.3030	0.0185
TCONS_00026625	1.0767	10.0724	0.0209
XR_001766358.1	1.0067	9.9754	0.0157
XR_001766718.1	0.9867	9.9464	0.0287
TCONS_00020612	0.8133	9.6677	0.0040
XR_001766449.1	0.6667	9.3808	0.0328
XR_001765311.1	0.5333	9.0589	0.0163
XR_001765691.1	0.3767	8.5571	0.0317
TCONS_00038794	1.0067	-9.9754	0.0188
TCONS_00015110	1.0067	-9.9754	0.0365
TCONS_00044926	1.0400	-10.0224	0.0348
XR_001766127.1	1.1467	-10.1632	0.0311
TCONS_00031037	1.1500	-10.1674	0.0410
XR_001765552.1	1.1567	-10.1758	0.0330
TCONS_00038591	1.2533	-10.2916	0.0097
XR_001766842.1	1.4767	-10.5281	0.0000
XR_001765500.1	1.4867	-10.5379	0.0221
XR_001765138.1	1.5200	-10.5699	0.0246
XR_001765856.1	1.5667	-10.6135	0.0224
TCONS_00002475	1.6833	-10.7171	0.0227
TCONS_00033478	1.7567	-10.7786	0.0401
TCONS_00035424	1.9700	-10.9440	0.0004
XR_001766337.1	2.5633	-11.3238	0.0350

TCONS_00004245	2.5800	-11.3332	0.0306
XR_001766606.1	2.6000	-11.3443	0.0023
XR_001766877.1	2.8100	-11.4564	0.0031
TCONS_00023581	2.9967	-11.5491	0.0067
TCONS_00004222	32.8900	-15.0054	0.0000
