# Supplementary material for: Systematic Characterization and Regulatory Role of lncRNAs in Asian Honey Bees Responding to Microsporidian Infestation
Source: Int J Mol Sci. 2023 Mar 20;24(6):5886. doi: 10.3390/ijms24065886 (PMC10058195; doi:10.3390/ijms24065886)
Supplement: Supplementary file 1 [file ijms-24-05886-s001.zip › Table S4.pdf]

Table S4. Top20 Class annotated by up-and down-stream genes of DElncRNAs in AcCK1 vs. AcT1 and AcCK2 vs. AcT2

Class	ID	Number	*P* value
Molecular Function	GO:0005548	3	4.02E-05
Molecular Function	GO:0005319	3	5.70E-05
Molecular Function	GO:0008134	2	0.000108605
Molecular Function	GO:0001882	11	0.000450299
Molecular Function	GO:0097367	11	0.000758995
Biological Process	GO:0015914	3	0.000132346
Biological Process	GO:0015748	3	0.000149198
Molecular Function	GO:0036094	11	0.001861245
Molecular Function	GO:0001883	10	0.002093988
Molecular Function	GO:0032549	10	0.002093988
Molecular Function	GO:0032550	10	0.002093988
Molecular Function	GO:0043169	11	0.002346846
Molecular Function	GO:0046872	8	0.002524953
Biological Process	GO:0006869	3	0.00030713
Molecular Function	GO:1901363	13	0.004095993
Biological Process	GO:0010876	3	0.000468413
Biological Process	GO:0015711	3	0.000630481
Molecular Function	GO:0043167	11	0.005298054
Biological Process	GO:0006720	2	0.001862705
Molecular Function	GO:0005548	3	4.02E-05

Class	ID	Number	*P* value
Biological Process	GO:0010467	7	0.001020445
Biological Process	GO:0010468	5	0.001248533
Biological Process	GO:0060255	5	0.002357304
Biological Process	GO:0019222	5	0.007524089
Biological Process	GO:0006401	1	0.010790256
Biological Process	GO:0043170	8	0.027995065
Biological Process	GO:0090305	1	0.032053859
Biological Process	GO:0090501	1	0.032053859
Biological Process	GO:0050789	6	0.048988468
Biological Process	GO:0065007	6	0.059831175
Biological Process	GO:0008152	10	0.06296173

Biological Process	GO:0009108	1	0.064629757
Biological Process	GO:0051188	1	0.074783827
Cellular Component	GO:0043231	2	0.09116591
Cellular Component	GO:0043227	2	0.097384436
Cellular Component	GO:0043229	2	0.152533793
Cellular Component	GO:0043226	2	0.15551484
Cellular Component	GO:0044424	2	0.246404954
Cellular Component	GO:0005622	2	0.247915479
Cellular Component	GO:0005623	2	0.32671665
