# Supplementary material for: Systematic Characterization and Regulatory Role of lncRNAs in Asian Honey Bees Responding to Microsporidian Infestation
Source: Int J Mol Sci. 2023 Mar 20;24(6):5886. doi: 10.3390/ijms24065886 (PMC10058195; doi:10.3390/ijms24065886)
Supplement: Supplementary file 1 [file ijms-24-05886-s001.zip › Table S5.pdf]

Table S5. Top20 pathways annotated by up-and down-stream genes of DElncRNAs in AcCK1 vs. AcT1 and AcCK2 vs. AcT2

Pathway	Up-steam genes	Down-steam genes	*P* value	koID
Apoptosis	4	0	3.31E-05	ko04210
Membrane Compartmentalization	3	0	0.000269	ko04670
Phototransduction-*Drosophila*	3	0	0.000727	ko04745
Gastric acid secretion	3	0	0.000833	ko04971
Platelet activation	3	0	0.001392	ko04611
Oxytocin signaling pathway	3	0	0.00263	ko04921
Thyroid Hormone signaling pathway	3	0	0.002805	ko04919
Thermogenesis	3	0	0.012954	ko04714
Basal Transcription Factor	1	2	0.000407	ko03022
Rap1 signaling pathway	3	0	0.004717	ko04015
Hippo signaling pathway-*Drosophila*	3	0	0.008163	ko04391
Tight Junction	3	0	0.001592	ko04520
Phagosome	3	0	0.002196	ko04145
Regulation of mitochondrial function	3	0	0.002196	ko04810
Focal adhesion	3	0	0.003579	ko04510
Adhesion junction	3	0	0.00577	ko04530
Gamma-aminobutyric acidergic synapses	0	2	0.014546	ko04727
Notch signaling pathway	0	2	0.009213	ko04330
FoxO signaling pathway	0	1	0.342028	ko04068
cAMP signaling pathway	0	1	0.329887	ko04024

Pathway	Up-steam genes	Down-steam genes	*P* value	koID
Metabolic pathways	1	3	0.6549822	ko01100
Purine metabolism	1	1	0.1708185	ko00230

RNA degradation	0	2	0.04876937	ko03018
RNA-transport	0	2	0.1157274	ko03013
Hippo signaling pathway-*Drosophila*	2	0	0.07744206	ko04391
Endocytosis	0	2	0.2075292	ko04144
MAPK signaling pathway-*Drosophila*	1	0	0.2937275	ko04013
Lysosome	1	0	0.3454199	ko04142
Cytochrome P450 drug metabolism	0	1	0.07145604	ko00982
Pentose and glucuronide conversion	0	1	0.08252695	ko00040
Folate biosynthesis	0	1	0.0912953	ko00790
Porphyrinoid and chlorophyll metabolism	0	1	0.09347519	ko00860
Drug metabolism-other enzyme	0	1	0.1464198	ko00983
Pyrimidine metabolism	0	1	0.3023136	ko00240
Biosynthesis of secondary metabolites	0	1	0.7853898	ko01110
Eukaryotic ribosome biogenesis	0	1	0.2474763	ko03008
Spliceosome	1	0	0.4630429	ko03040
Two-component systems	0	1	0.07145604	ko02020
Pantothenate and coenzyme-A biosynthesis	0	1	0.0512152	ko00770
Cytochrome P450 exogenous substance metabolism	0	1	0.07811338	ko00980
