# Supplementary material for: Systematic Characterization and Regulatory Role of lncRNAs in Asian Honey Bees Responding to Microsporidian Infestation
Source: Int J Mol Sci. 2023 Mar 20;24(6):5886. doi: 10.3390/ijms24065886 (PMC10058195; doi:10.3390/ijms24065886)
Supplement: Supplementary file 1 [file ijms-24-05886-s001.zip › Table S6.pdf]

Table S6. MRNAs expressed by DElncRNAs in the AcCK1 vs AcT1 and AcCK2 vs AcT2 comparison groups

lncRNA-id	mRNA-id
XR_001767038.1	XM_017062550.1,XM_017057235.1
TCONS_00047233	XM_017050788.1,XM_017067098.1
XR_001766127.1	XM_017063176.1,XM_017053534.1
XR_001765163.1	XM_017058679.1,XM_017065924.1
TCONS_00029392	XM_017063756.1,XM_017058121.1
XR_001765315.1	XM_017056184.1,XM_017051511.1
TCONS_00006106	XM_017056732.1,XM_017062597.1
TCONS_00029812	XM_017063552.1,XM_017051511.1
XR_001765926.1	XM_017061716.1,XM_017058706.1,XM_017052428.1,XM_017058103.1,XM_017050493.1,XM_017056875.1,XM_017059240.1,XM_017052005.1,XM_017060925.1,XM_017052018.1,XM_017063901.1,XM_017056763.1,XM_017067401.1,XM_017055286.1,XM_017057669.1,XM_017053748.1,XM_017060075.1,XM_017063149.1,XM_017058242.1,XM_017049871.1,XM_017052722.1,XM_017064871.1,XM_017052762.1,XM_017059301.1,XM_017062572.1,XM_017057531.1,XM_017053027.1,XM_017052276.1,XM_017050864.1,XM_017049818.1,XM_017054405.1,XM_017064495.1,XM_017058468.1,XM_017059346.1,XM_017056401.1,XM_017064528.1,XM_017054408.1,XM_017065909.1,XM_017052763.1,XM_017050928.1,XM_017049655.1,XM_017057380.1,XM_017063543.1,XM_017052879.1,XM_017061117.1,XM_017061958.1,XM_017049610.1,XM_017062921.1,XM_017061240.1,XM_017058682.1,XM_017054355.1,XM_017062428.1,XM_017064038.1,XM_017065829.1,XM_017049283.1,XM_017058844.1,XM_017067284.1,XM_017059142.1,XM_017059807.1,XM_017053449.1,XM_017053177.1,XM_017057014.1,XM_017060643.1,XM_017054603.1,XM_017054797.1,XM_017053248.1,XM_017065933.1,XM_017051430.1,XM_017061555.1,XM_017062801.1,XM_017054910.1,XM_017065356.1,XM_017061093.1,XM_017057304.1,XM_017051059.1,XM_017063902.1,XM_017051674.1,XM_017063435.1,XM_017064577.1,XM_017050657.1,XM_017049608.1,XM_017053244.1,XM_017057001.1,XM_017049772.1,XM_017062465.1,XM_017066249.1,XM_017053951.1,XM_017053018.1,XM_017053347.1,XM_017064689.1
TCONS_00004253	XM_017067450.1,XM_017062339.1
XR_001767044.1	XM_017062449.1,XM_017063786.1
TCONS_00012188	XM_017066294.1,XM_017061562.1
XR_001765299.1	XM_017054323.1,XM_017049733.1

TCONS_00032459	XM_017060234.1,XM_017051337.1
TCONS_00017999	XM_017054787.1,XM_017062989.1
XR_001765180.1	XM_017052676.1,XM_017062518.1
TCONS_00022015	XM_017056849.1,XM_017052871.1
TCONS_00019291	XM_017056927.1,XM_017062310.1
TCONS_00019419	XM_017063722.1,XM_017064407.1
XR_001765320.1	XM_017060131.1,XM_017057242.1
TCONS_00041487	XM_017054863.1,XM_017061269.1
XR_001766556.1	XM_017064469.1,XM_017065979.1
TCONS_00024312	XM_017067475.1,XM_017062900.1
TCONS_00009793	XM_017054930.1,XM_017058366.1
XR_001766050.1	XM_017063552.1,XM_017063786.1
TCONS_00009787	XM_017062667.1,XM_017058479.1
TCONS_00004326	XM_017058091.1,XM_017051854.1
TCONS_00004057	XM_017053518.1,XM_017056037.1
TCONS_00034859	XM_017060165.1,XM_017058989.1
TCONS_00031168	XM_017054654.1,XM_017056190.1
TCONS_00036996	XM_017056253.1,XM_017054885.1
TCONS_00024562	XM_017058286.1,XM_017054559.1
XR_001766023.1	XM_017056248.1,XM_017063786.1
XR_001765162.1	XM_017064887.1,XM_017050832.1
XR_001767030.1	XM_017053281.1,XM_017058121.1
TCONS_00044021	XM_017054581.1,XM_017062578.1
TCONS_00047756	XM_017062963.1,XM_017050832.1
XR_001766711.1	XM_017061726.1,XM_017057376.1
XR_001767155.1	XM_017064097.1,XM_017062945.1
TCONS_00031567	XM_017050604.1,XM_017059567.1
XR_001765824.1	XM_017059391.1,XM_017058008.1
XR_001766919.1	XM_017063532.1,XM_017050831.1
TCONS_00047254	XM_017057080.1,XM_017053643.1
TCONS_00028563	XM_017049925.1,XM_017061159.1
XR_001765757.1	XM_017063556.1,XM_017060997.1
XR_001766833.1	XM_017059863.1,XM_017051824.1
TCONS_00012932	XM_017052415.1,XM_017058008.1
TCONS_00044420	XM_017065214.1,XM_017061581.1
TCONS_00040767	XM_017054648.1,XM_017050831.1
XR_001765880.1	XM_017050770.1,XM_017057346.1
TCONS_00032996	XM_017050379.1,XM_017049020.1
XR_001766455.1	XM_017055784.1,XM_017053101.1

XR_001765600.1	XM_017057882.1,XM_017062468.1
XR_001767039.1	XM_017059731.1,XM_017060034.1
XR_001765130.1	XM_017060601.1,XM_017062450.1
TCONS_00021820	XM_017049986.1,XM_017059863.1
TCONS_00022174	XM_017051805.1,XM_017063096.1
TCONS_00016200	XM_017055902.1,XM_017050245.1
XR_001765805.1	XM_017050140.1,XM_017052983.1
TCONS_00042235	XM_017056034.1,XM_017067348.1
TCONS_00026623	XM_017051595.1,XM_017053918.1
TCONS_00019289	XM_017056643.1,XM_017063790.1
XR_001765993.1	XM_017061599.1,XM_017061685.1
XR_001764971.1	XM_017055966.1,XM_017058897.1
TCONS_00011721	XM_017049515.1,XM_017049439.1
TCONS_00046801	XM_017050066.1,XM_017056619.1
XR_001766966.1	XM_017060689.1,XM_017056444.1
TCONS_00037364	XM_017052728.1,XM_017054728.1
TCONS_00038004	XM_017059774.1,XM_017055640.1
XR_001765515.1	XM_017066287.1,XM_017066634.1
TCONS_00031155	XM_017058159.1,XM_017049376.1
TCONS_00001436	XM_017054632.1,XM_017059980.1
XR_001765500.1	XM_017049771.1,XM_017057863.1
XR_001766403.1	XM_017064791.1,XM_017061032.1
XR_001766879.1	XM_017060563.1,XM_017062832.1
XR_001765115.1	XM_017065470.1,XM_017065472.1
XR_001766607.1	XM_017062842.1,XM_017062450.1
XR_001765791.1	XM_017057993.1,XM_017063532.1
TCONS_00006288	XM_017061241.1,XM_017065883.1

lncRNA-id	mRNA-id
XR_001767038.1	XM_017062550.1,XM_017057235.1
TCONS_00047233	XM_017050788.1,XM_017067098.1
XR_001766127.1	XM_017063176.1,XM_017053534.1
XR_001765163.1	XM_017058679.1,XM_017065924.1
TCONS_00029392	XM_017063756.1,XM_017058121.1
XR_001765315.1	XM_017056184.1,XM_017051511.1
TCONS_00006106	XM_017056732.1,XM_017062597.1
TCONS_00029812	XM_017063552.1,XM_017051511.1
XR_001765926.1	XM_017061716.1,XM_017058706.1,XM_017052428.1,XM_017058103.1,XM_017050493.1,XM_017056875.1,XM_017059240.1,XM_017052005.1,XM_017060925.1,XM_017052018.1,

	XM_017063901.1,XM_017056763.1,XM_017067401.1,XM_017055286.1,XM_017057669.1,XM_017053748.1,XM_017060075.1,XM_017063149.1,XM_017058242.1,XM_017049871.1,XM_017052722.1,XM_017064871.1,XM_017052762.1,XM_017059301.1,XM_017062572.1,XM_017057531.1,XM_017053027.1,XM_017052276.1,XM_017050864.1,XM_017049818.1,XM_017054405.1,XM_017064495.1,XM_017058468.1,XM_017059346.1,XM_017056401.1,XM_017064528.1,XM_017054408.1,XM_017065909.1,XM_017052763.1,XM_017050928.1,XM_017049655.1,XM_017057380.1,XM_017063543.1,XM_017052879.1,XM_017061117.1,XM_017061958.1,XM_017049610.1,XM_017062921.1,XM_017061240.1,XM_017058682.1,XM_017054355.1,XM_017062428.1,XM_017064038.1,XM_017065829.1,XM_017049283.1,XM_017058844.1,XM_017067284.1,XM_017059142.1,XM_017059807.1,XM_017053449.1,XM_017053177.1,XM_017057014.1,XM_017060643.1,XM_017054603.1,XM_017054797.1,XM_017053248.1,XM_017065933.1,XM_017051430.1,XM_017061555.1,XM_017062801.1,XM_017054910.1,XM_017065356.1,XM_017061093.1,XM_017057304.1,XM_017051059.1,XM_017063902.1,XM_017051674.1,XM_017063435.1,XM_017064577.1,XM_017050657.1,XM_017049608.1,XM_017053244.1,XM_017057001.1,XM_017049772.1,XM_017062465.1,XM_017066249.1,XM_017053951.1,XM_017053018.1,XM_017053347.1,XM_017064689.1
TCONS_00004253	XM_017067450.1,XM_017062339.1
XR_001767044.1	XM_017062449.1,XM_017063786.1
TCONS_00012188	XM_017066294.1,XM_017061562.1
XR_001765299.1	XM_017054323.1,XM_017049733.1
TCONS_00032459	XM_017060234.1,XM_017051337.1
TCONS_00017999	XM_017054787.1,XM_017062989.1
XR_001765180.1	XM_017052676.1,XM_017062518.1
TCONS_00022015	XM_017056849.1,XM_017052871.1
TCONS_00019291	XM_017056927.1,XM_017062310.1
TCONS_00019419	XM_017063722.1,XM_017064407.1
XR_001765320.1	XM_017060131.1,XM_017057242.1
TCONS_00041487	XM_017054863.1,XM_017061269.1
XR_001766556.1	XM_017064469.1,XM_017065979.1
TCONS_00024312	XM_017067475.1,XM_017062900.1
TCONS_00009793	XM_017054930.1,XM_017058366.1
XR_001766050.1	XM_017063552.1,XM_017063786.1
TCONS_00009787	XM_017062667.1,XM_017058479.1
TCONS_00004326	XM_017058091.1,XM_017051854.1
TCONS_00004057	XM_017053518.1,XM_017056037.1
TCONS_00034859	XM_017060165.1,XM_017058989.1

TCONS_00031168	XM_017054654.1,XM_017056190.1
TCONS_00036996	XM_017056253.1,XM_017054885.1
TCONS_00024562	XM_017058286.1,XM_017054559.1
XR_001766023.1	XM_017056248.1,XM_017063786.1
XR_001765162.1	XM_017064887.1,XM_017050832.1
XR_001767030.1	XM_017053281.1,XM_017058121.1
TCONS_00044021	XM_017054581.1,XM_017062578.1
TCONS_00047756	XM_017062963.1,XM_017050832.1
XR_001766711.1	XM_017061726.1,XM_017057376.1
XR_001767155.1	XM_017064097.1,XM_017062945.1
TCONS_00031567	XM_017050604.1,XM_017059567.1
XR_001765824.1	XM_017059391.1,XM_017058008.1
XR_001766919.1	XM_017063532.1,XM_017050831.1
TCONS_00047254	XM_017057080.1,XM_017053643.1
TCONS_00028563	XM_017049925.1,XM_017061159.1
XR_001765757.1	XM_017063556.1,XM_017060997.1
XR_001766833.1	XM_017059863.1,XM_017051824.1
TCONS_00012932	XM_017052415.1,XM_017058008.1
TCONS_00044420	XM_017065214.1,XM_017061581.1
TCONS_00040767	XM_017054648.1,XM_017050831.1
XR_001765880.1	XM_017050770.1,XM_017057346.1
TCONS_00032996	XM_017050379.1,XM_017049020.1
XR_001766455.1	XM_017055784.1,XM_017053101.1
XR_001765600.1	XM_017057882.1,XM_017062468.1
XR_001767039.1	XM_017059731.1,XM_017060034.1
XR_001765130.1	XM_017060601.1,XM_017062450.1
TCONS_00021820	XM_017049986.1,XM_017059863.1
TCONS_00022174	XM_017051805.1,XM_017063096.1
TCONS_00016200	XM_017055902.1,XM_017050245.1
XR_001765805.1	XM_017050140.1,XM_017052983.1
TCONS_00042235	XM_017056034.1,XM_017067348.1
TCONS_00026623	XM_017051595.1,XM_017053918.1
TCONS_00019289	XM_017056643.1,XM_017063790.1
XR_001765993.1	XM_017061599.1,XM_017061685.1
XR_001764971.1	XM_017055966.1,XM_017058897.1
TCONS_00011721	XM_017049515.1,XM_017049439.1
TCONS_00046801	XM_017050066.1,XM_017056619.1
XR_001766966.1	XM_017060689.1,XM_017056444.1
TCONS_00037364	XM_017052728.1,XM_017054728.1
TCONS_00038004	XM_017059774.1,XM_017055640.1
XR_001765515.1	XM_017066287.1,XM_017066634.1
TCONS_00031155	XM_017058159.1,XM_017049376.1
TCONS_00001436	XM_017054632.1,XM_017059980.1
XR_001765500.1	XM_017049771.1,XM_017057863.1

XR_001766403.1	XM_017064791.1,XM_017061032.1
XR_001766879.1	XM_017060563.1,XM_017062832.1
XR_001765115.1	XM_017065470.1,XM_017065472.1
XR_001766607.1	XM_017062842.1,XM_017062450.1
XR_001765791.1	XM_017057993.1,XM_017063532.1
TCONS_00006288	XM_017061241.1,XM_017065883.1
