# Supplementary material for: Systematic Characterization and Regulatory Role of lncRNAs in Asian Honey Bees Responding to Microsporidian Infestation
Source: Int J Mol Sci. 2023 Mar 20;24(6):5886. doi: 10.3390/ijms24065886 (PMC10058195; doi:10.3390/ijms24065886)
Supplement: Supplementary file 1 [file ijms-24-05886-s001.zip › Table S7.pdf]

**Table S7.** Targeting relationships among DElncRNAs, DEmiRNAs and DEmRNAs in AcCK1 vs. AcT1 and AcCK2 vs. AcT2 comparison group

| DElncRNAID | DEmiRNAID | DEmRNAID | Binding Free Energy of miRNA and lncRNA | Binding Free Energy of miRNA and RNA | *P* value |
|---|---|---|---|---|---|
| XR_001766455.1 | miR-8462-x | XM_017052046.1 | -16.4 | -14.2 | 5.27E-05 |
| XR_001766455.1 | miR-8462-x | XM_017055319.1 | -14.9 | -14.2 | 0.00043366 |
| TCONS_00032996 | miR-6717-x | XM_017064838.1 | -16.2 | -22.5 | 0.00036257 |
| TCONS_00032996 | miR-6717-x | XM_017053515.1 | -26.1 | -22.5 | 0.00596561 |
| TCONS_00032996 | miR-6717-x | XM_017063775.1 | -18.8 | -22.5 | 0.00010488 |
| TCONS_00032996 | miR-252-y | XM_017056322.1 | -20.5 | -22.9 | 0.00597207 |
| TCONS_00032996 | miR-252-y | XM_017067320.1 | -20.1 | -22.9 | 0.00341161 |
| TCONS_00032996 | miR-252-y | XM_017053959.1 | -20.6 | -22.9 | 0.00442765 |
| TCONS_00032996 | miR-252-y | XM_017049653.1 | -21.8 | -22.9 | 0.00371617 |
| TCONS_00032996 | miR-252-y | XM_017057307.1 | -23.4 | -22.9 | 6.16E-05 |
| TCONS_00022015 | miR-1-x | XM_017051758.1 | -21 | -19.3 | 3.23E-06 |
| TCONS_00022015 | miR-1-x | XM_017062549.1 | -22.6 | -19.3 | 0.00955882 |
| TCONS_00022015 | miR-598-y | XM_017064722.1 | -25.8 | -21.7 | 1.75E-08 |
| TCONS_00022015 | miR-598-y | XM_017050546.1 | -18.3 | -21.7 | 0.00099881 |
| TCONS_00022015 | miR-598-y | XM_017051858.1 | -28.2 | -21.7 | 0.00250497 |
| TCONS_00022015 | miR-598-y | XM_017057157.1 | -19.7 | -21.7 | 0.00205145 |
| TCONS_00022015 | miR-598-y | XM_017062062.1 | -37.6 | -21.7 | 0.00368195 |
| XR_001765791.1 | miR-6313-y | XM_017051268.1 | -18.1 | -18.8 | 0.00057396 |
| TCONS_00028563 | miR-598-y | XM_017059863.1 | -24.9 | -28.3 | 0.00307432 |
| TCONS_00028563 | miR-598-y | XM_017051858.1 | -28.2 | -28.3 | 0.00578352 |
| TCONS_00028563 | miR-598-y | XM_017064421.1 | -21 | -28.3 | 0.00294688 |
| XR_001766607.1 | miR-676-y | XM_017060338.1 | -18.3 | -28.4 | 0.00124322 |
| XR_001766607.1 | miR-676-y | XM_017060344.1 | -18.3 | -28.4 | 8.58E-06 |
| TCONS_00016200 | miR-676-y | XM_017054112.1 | -17.9 | -22.4 | 0.00222314 |
| XR_001765130.1 | miR-676-y | XM_017065309.1 | -24.2 | -18.8 | 0.00016853 |
| XR_001765130.1 | miR-676-y | XM_017060344.1 | -18.3 | -18.8 | 1.38E-05 |
| TCONS_00044021 | miR-965-x | XM_017059863.1 | -23.4 | -20.3 | 0.00324607 |
| TCONS_00044021 | miR-965-x | XM_017055968.1 | -17.3 | -20.3 | 0.00342744 |

| DElncRNAID | DEmiRNAID | DEmRNAID | Binding Free Energy of miRNA and lncRNA | Binding Free Energy of miRNA and RNA | *P* value |
|---|---|---|---|---|---|
| XR_001766995.1 | novel-m0019-5p | XM_017049683.1 | -17.3 | -23.7 | 0.000109 |
| XR_001766995.1 | novel-m0019-5p | XM_017059913.1 | -26.3 | -23.7 | 0.000909 |
| XR_001766995.1 | novel-m0019-5p | XM_017060060.1 | -19.3 | -23.7 | 0.000163 |
| TCONS_00007982 | novel-m0003-3p | XM_017059841.1 | -20.7 | -19.9 | 0.00034 |
| TCONS_00007982 | novel-m0003-3p | XM_017066597.1 | -20 | -19.9 | 7.67E-06 |
| XR_001765691.1 | novel-m0003-3p | XM_017059841.1 | -20.7 | -19.3 | 5.26E-06 |
| XR_001765691.1 | novel-m0003-3p | XM_017066597.1 | -20 | -19.3 | 0 |
| TCONS_00035424 | miR-6313-y | XM_017063363.1 | -21 | -20.6 | 0.002259 |
| XR_001765313.1 | miR-3720-x | XM_017064098.1 | -18.1 | -20.8 | 0.000503 |
| XR_001766943.1 | miR-60-y | XM_017063763.1 | -17.9 | -15.3 | 9.32E-05 |
