# Supplementary material for: Systematic Characterization and Regulatory Role of lncRNAs in Asian Honey Bees Responding to Microsporidian Infestation
Source: Int J Mol Sci. 2023 Mar 20;24(6):5886. doi: 10.3390/ijms24065886 (PMC10058195; doi:10.3390/ijms24065886)
Supplement: Supplementary file 1 [file ijms-24-05886-s001.zip › Table S8.pdf]

Table S8. Sequences of primers for RT-qPCR validation of DElncRNAs

LncRNA ID	Primer sequence (5′-3′)
XR_001765880.1	F: AACGAATACGACCGAACTAA<br>R: CAACTACATTGTCCACCAGA
XR_001766754.1	F: AAATGGACTTACAATGCGAGAA<br>R: GTGGGTCAACTAAACGGAAA
XR_001766833.1	F: TGAAACTGTGGTGGAACT<br>R: ATTGGAGGATAACTAAGGA
XR_001766919.1	F: ACCTCGGTGCTCGTAG<br>R: TCCCAGTCCTGGCTTA
TCONS_00030276	F: GCGGATAATGACGATGGA<br>R: GGCTCTTGGTAGACACTTGC
XR_001765676.1	F: CGGACAGACAGAGATTCCAAC<br>R: TTCACACGAAGGCAGCGTA
XR_001765714.1	F: CGTTACAACAGAGTCAACAGGG<br>R: GTGCGGTCAAAGCGATTA
XR_001766526.1	F: AGCAGTGAATGTAGCCAACA<br>R: TTTCTGGTTCGCCTTGAC
TCONS_00004123	F: GAAGGGAAAGGAAAGAACG<br>R: GGAAGTTGGTAGGAGAAGGAT
TCONS_00025811	F: CGCAATCCGTCTCAAGAAG<br>R: TAGCAGTGGCAGCCGTAAT
XR_001767039.1	F: CGTGTTCCACTACCACCTCT<br>R: CCCGCCATTCGTTATCTA
XR_001765857.1	F: CTACGAATGTGCCTCATCC<br>R: CGACCTCTAACTACGAGTCTGC
actin	F: GGTTGTTGATAGTGGAGATGG<br>R: CACGACCAGCAATAGGAAT
