# Supplementary figures and images for: Systematic Characterization and Regulatory Role of lncRNAs in Asian Honey Bees Responding to Microsporidian Infestation
Source: Int J Mol Sci. 2023 Mar 20;24(6):5886. doi: 10.3390/ijms24065886 (PMC10058195; doi:10.3390/ijms24065886)

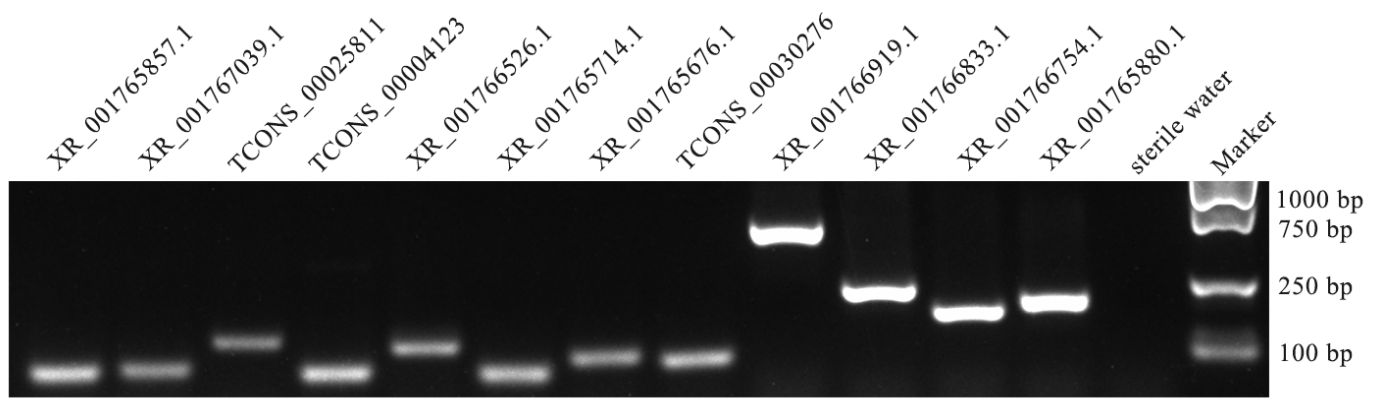

Figure S2. Agarose electrophoresis validation of RT-qPCR primer specificity

Supplement: Supplementary file 1 [file ijms-24-05886-s001.zip › Figure S2.pdf]
